# Diversity, Physiochemical and Phylogenetic Analyses of Bacteria Isolated from Various Drinking Water Sources

**DOI:** 10.12669/pjms.333.12347

**Published:** 2017

**Authors:** Neveen H. Eid, Huda A. Al Doghaither, Taha A. Kumosani, Munazza Gull

**Affiliations:** 1 Neveen H. Eid, Biochemistry Department, Faculty of Science, King Abdulaziz University, P.O. Box 42805, Jeddah, Saudi Arabia; 2 Dr. Huda A. Al Doghaither, Biochemistry Department, Faculty of Science, King Abdulaziz University, P.O. Box 42805, Jeddah, Saudi Arabia; 3 Prof. Dr. Taha A. Kumosani, Biochemistry Department, Faculty of Science, King Abdulaziz University, P.O. Box 42805, Jeddah, Saudi Arabia; 4 Dr. Munazza Gull, Biochemistry Department, Faculty of Science, King Abdulaziz University, P.O. Box 42805, Jeddah, Saudi Arabia

**Keywords:** Drinking water, 16SrRNA, *Bacillus* bacteria, Human disease, Saudi Arabia

## Abstract

**Objective:**

To evaluate the indigenous bacterial strains of drinking water from the most commercial water types including bottled and filtered water that are currently used in Saudi Arabia.

**Methods:**

Thirty randomly selected commercial brands of bottled water were purchased from Saudi local markets. Moreover, samples from tap water and filtered water were collected in sterilized glass bottles and stored at 4°C. Biochemical analyses including pH, temperature, lactose fermentation test (LAC), indole test (IND), methyl red test (MR), Voges-Proskauer test (VP), urease test (URE), catalase test (CAT), aerobic and anaerobic test (Ae/An) were measured. Molecular identification and comparative sequence analyses were done by full length 16S rRNA gene sequences using gene bank databases and phylogenetic trees were constructed to see the closely related similarity index between bacterial strains.

**Results:**

Among 30 water samples tested, 18 were found positive for bacterial growth. Molecular identification of four selected bacterial strains indicated the alarming presence of pathogenic bacteria *Bacillus spp*. in most common commercial types of drinking water used in Saudi Arabia.

**Conclusion:**

The lack of awareness about good sanitation, poor personal hygienic practices and failure of safe water management and supply are the important factors for poor drinking water quality in these sources, need to be addressed.

## INTRODUCTION

The quality of water is important to the health, social and economic wellbeing of people. It is important to test the suitability of the quality of water for its use as drinking water. Water that looks potable can contain bacterial contamination, which are not visible to naked eye and cannot be detected by smell, taste and sight. Due to anthropogenic interventions, the water is getting polluted and thus causing negative effects on human health and natural equilibrium. The pollution in water sources has become one of the primary problems worldwide.^1^,^2^

Bacteria are one of the major contaminants of water.^3^ They have been reported to persist even in the extreme environmental conditions and oligotrophic conditions. Moreover, many of the bacterial species have the ability to make resistant survival structures.^4^ The aim of the current research was to investigate the microbial contaminations and compare their diversity in various drinking water sources. The detection was done by biochemical screening assays and phylogenetic of 16S rRNA gene sequences based diagnostic polymerase chain reaction (PCR) for molecular identification.

## METHODS

### Water samples collection and physiochemical characterization

Thirty samples of water were used in this study. Of these, 23 brands (76.6%) of bottled water were obtained from local markets in Jeddah, Saudi Arabia with their source or production site in Saudi Arabia, 4 brands (13.3%) were imported (manufactured by foreign companies but used in Saudi Arabia), and 10% were from local tap water and filtered water collected from Jeddah city, Saudi Arabia. Commercial bottled water samples were collected in sterile plastic bottles. The sizes of the bottles ranged from 250ml-10 L and were stored immediately in their original closed plastic containers at 4°C until analysis was made. Tap water and filtered water samples were collected in sterilized glass bottles and stored at 4°C for 24 hour. The pH and temperature of all samples were measured at the same time and after one hour of sample collection The pH of all water samples was measured using pH PAL high accuracy electrochemistry test pen. The cap was removed and the tester was calibrated with buffer solution at pH 7, then the tester was dipped up to immersed level in water sample and after one minute, results were recorded. The temperature of the water samples was measured using electronic, digital thermometer (Quartz Oregon Scientific, USA).

### Isolation and purification of bacterial isolates

Isolation of bacteria was performed on Tryptic Soya Agar (TSA) (HIMEDIA) medium using spread plate technique. The TSA plates were incubated for 24 to 48 h at 37°C, after inoculation with 100 μl of the sample and subsequently were observed for bacterial growth and isolation. In a given plate, all the isolates with differential colony morphology were selected and kept in slant at 4°C.

### Gram stainingand biochemical analyses

The bacterial isolates were purified using the streak plate method and characterized by Gram PVP kit (QCA). Seven biochemical tests were performed for the preliminary identification of bacteria according to Bergey’s Manual and ABIS 7 online software. The principal biochemical tests performed for this purpose include, lactose fermentation test (LAC), indole test (IND), methyl red test (MR), Voges-Proskauer test (VP), urease test (URE), catalase test (CAT), aerobic and anaerobic test (Ae/An).

### Molecular identification of selected bacteria by 16S rRNA gene sequencing

Genomic DNA was extracted using a GeneJET genomic DNA purification kit (Thermo Scientific, Waltham, MA, USA) following the manufacture’s instructions. The extracted DNA was detected using agarose gel electrophoresis and visualized by ethidium bromide dye. The complete 1.5 Kb 16S rRNA region was amplified using Go Taq Green Master mix (Promega) and primers P1 (100 pmol/μl) as forward primer (MACRO GEN) and P6 (100 pmol/μl) as reverse primer (MACRO GEN).

### Forward primer

(5`- CGGGATCCAGAGTTTGATCCTGGTCAGAACGAACGCT-3’).

### Reverse primer

(5´CGGGATCCTACGGCT ACC TTGTT AC GACTTCACCCC-3’).

### PCR mixture preparation and conditions

The reaction mixture (25 μl) was prepared by adding 2 μl forward primer, 2 μl reverse primer, 6 μl DNA, 12.5 μl master mix solution and 2.2 μl water free nuclease for full length 16S rRNA gene amplification and initially was denatured at 94°C for 2 min followed by 30 cycles consisting of denaturation at 94°C for 60 s, primer annealing at 55°C for 60s and primer extension at 72°C for 3 min and a final extension at 72°C for 10 min using multigene thermal cycler. PCR products were analyzed through gel electrophoresis.

### Gene cloning

PCR products were cloned into 2886 base pair (bp) pTZ57R/T vector by using InsTAclone PCR cloning kit (Thermo Scientific). This vector carries a gene for ampicillin resistance and the lac Z gene fragment to provide blue/white selection. The plasmid purified products were sequenced using M13 primer.

### Comparative sequence analysis

Gene sequences were analyzed by comparing them with known 16S rRNA sequences using others in the Gene Bank databases by the NCBI BLAST algorithm (http://www.ncbi.n1m.nih.gov/blast/Blast.cgi), to find the closest match in Gene Bank, EMBL, DDBJ, and PDB sequence data. Phylogenetic tree was created by using MEGA4.1 software package using the neighbor-joining method with automation correction.

## RESULTS

From 30 different water samples tested for water quality, 18 samples were found positive for bacterial growth with 28 different types of bacterial colonies.

### Physicochemical parameters

The temperaturesof water samples ranged between 19-20ºC and the pH of water samples ranged from 6.8 to 8.

### Gram staining and biochemical analysis

From 18 water samples with positive bacterial growth, 18 different colonies were selected to be tested for Gram staining andbiochemical tests. The results are shown in Table-I.

**Table-I:** Gram staining and biochemical analysis of bacterial samples isolated from various drinking water sources.

*Serial No*	*Bacterial samples*	*Gram staining*	*LAC Test*	*IND test*	*MR test*	*VP test*	*URE Test*	*CAT Test*	*Ae/An test*
1	1	+	+	-	+	+	-	+	+
2	2	+	+	+	+	+	-	+	+
3	3^a^	-	-	+	-	-	-	+	+
4	3^b^	-	+	+	-	-	-	-	+
5	6^a^	+	+	-	+	+	-	+	+
6	6^b^	+	+	-	+	+	+	+	+
7	7^a^	+	+	-	+	+	-	+	+
8	8	-	+	+	-	-	-	+	+
9	10	-	+	-	-	-	+	-	+
10	11	+	+	+	+	+	+	+	+
11	13	+	+	-	-	-	-	+	+
12	19^a^	+	+	-	+	+	-	+	+
13	19^b^	-	+	+	-	+	-	+	+
14	19^c^	-	-	-	-	-	-	+	+
15	20^a^	-	+	-	+	+	-	+	+
16	20^b^	+	+	-	+	+	-	+	+
17	22	-	-	+	-	+	-	-	+
18	23	+	+	-	+	+	-	+	+

(+) means positive results, (-) means negative results. (LAC): Lactose fermentation test, (IND): Indole test, (MR): Methyl red test, (VP): Voges-Proskauer test, (URE): Urease test, (CAT): Catalase test, (Ae/An): Aerobic and anaerobic test.

### Selection of potent bacteria for molecular identification

From 18 bacterial isolates, 4 bacterial isolates (1, 6a, 7a, and 20b) were selected for DNA sequencing randomly on the basis of Colony morphology, biochemical characterization results, and most often occurence.

### DNA extraction of potent bacteria

The DNA extracts were detected by gel electrophoresis.

### Amplification of full length 16S rRNA gene

**Fig.1:**
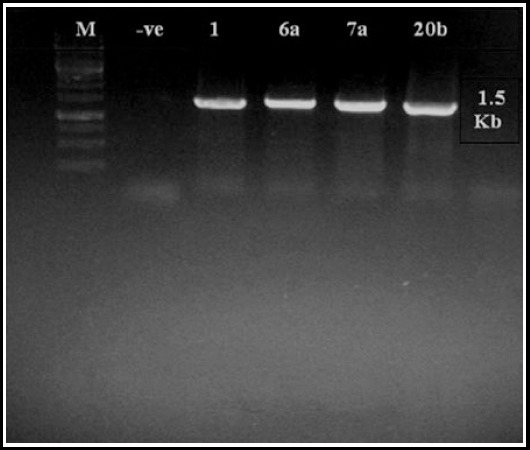
PCR amplification showed 1.5 Kb band size according to 16S rRNA gene. M Represents molecular marker 1 Kb, -ve: Negative control. Lane 3-6 represents PCR products amplification of bacterial samples 1, 6a, 7a, and 20b.

Primers, P1 and P6 amplified 1.5 kb fragment of 16S rRNA gene when the total genomic DNA of bacterial isolates was used as a template in PCR (Fig.1).

### Rapid plasmid miniprep

**Fig.2:**
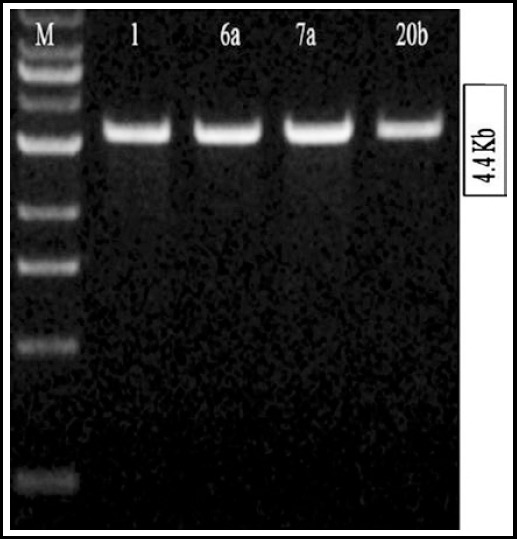
Isolation and purification of plasmid for full length DNA sequencing analysis of 16S rRNA gene. M Represents molecular marker 1 Kb, Plasmids were isolated and purified for four samples 1, 6a, 7a, and 20b. Lane 2-5 represents purified Plasmids from bacterial samples 1, 6a, 7a, and 20b.

The isolated plasmid, containing full length 16S rRNA gene cloned in TA cloning vector, was run at 1% agarose gel prepared in 0.5X TBE buffer. The results showed that the clone size was in the range of 4300-4400 bp (Fig.2).

### Sequence of 16S rRNA gene of bacterial strain and blast analysis of 16S rRNA gene fragments

The full length 16S rRNA gene’s nucleotide sequence of 1, 6a, 7a, and 20b was determined by automated sequencer. The nucleotide sequence was aligned and compared with standard strains in Blast. Homology results for nucleotide sequence of 16S rRNA gene of 1, 6a, 7a and 20b, and the submission of nucleotide sequence of 1, 6a, 7a, and 20b to gene bank provided the accession number for these bacterial strains. Phylogenetic trees were created by using MEGA4.1 software package using the neighbor-joining methodwith automation correction. The sequence of 16S rRNA genes revealed that the closest phylogenetic neighbor of one and 6a is *Bacillus cerusus*, with 99% similarity of nucleotide sequence. 16S rRNA gene sequence of the strain number 7a clearly indicated that the strain belonged to the family *Bacillaceae* most closely related to *Bacilli sp*. with 94% nucleotide sequence similarity. On these bases, strain 20b was identified as *Bacillusthuringiensis* with 98% similarity of nucleotide sequence (Table-II).

**Table-II:** Molecular identification on the basis of 16S rRNA gene sequence analysis. Bacterial sequences producing significant alignment with accession numbers.

*Serial Numbers*	*Bacterial samples*	*Description*	*Sequence ID*	*Nearest Relatives*	*Identity %*
1	1	Bacillus cerusus	KM 051083.1	Bacillus cerusus ATCC 14579	99%
2	6a	Bacillus cerusus	NR 113990.1	Bacillus cerusus NBRC 101232	99%
3	7a	Bacillus sp.	JX 56656.1	Bacillus sp. 6139	94%
4	20b	Bacillus thuringiensis	KF 150391.1	Bacillus thuringiensis JN 106	98%

## DISCUSSION

Thirty samples of different drinking water sources available and used in Saudi Arabia were analyzed, 18 samples were found contaminated with bacteria. The bacterial isolates were analyzed for further characterization on the basis of their colonies, morphology and growth pattern diversity. High bacterial diversity found in this study might be due to the multiple chances of contaminations from the environment, due to lack of care and awareness.

In the current study, results showed that the temperature of drinking water ranged between 19ºC-20ºC. A similar study by Hussian et al.^5^ reported a temperature ranges of 25.5ºC to 29.5ºC. The temperature ranges of water samples reported in this study is presumably. Due to sunlight intensity, the temperature elevates from 17ºC in cold season to 50ºC in summer season.^6^,^7^ The pH of water samples was found between 6.8 to 8. The huge variation in pH is dependent on the type of bottled water and manufacturing conditions.

Moreover, the data showed that most of the drinking water sources are contaminated with *Bacillus spp*. particularly and with other pathogenic bacteria generally. The bacterial species isolated were mostly belongs to family of bacilli and are Gram positive. The species isolated in this study also included *Bacillus cereus, Bacillus thuringiensis* and *Bacillus sp*. which are the known indicators of water contamination.^8^-^10^

Biochemical analysis of the genus Bacillus gave negative results for indole, citrate, oxidase, and urease and positive results for methyl red Voges-Proskaeur, catalase, and gelatinase tests. In many previous researches, biochemical tests were used to identify the bacterial strains tentatively and indicating their pathogenic potential for various diseases. Hussain and his colleagues recorded a variety of techniques that have been reported so far to evaluate the ecology of bacteria in drinking water.^11^,^12^

Qualitative and quantitative composition of pathogenic bacteria in water samples was probably due to insufficient preventive measures in drinking water sector. The difference in the quantitative frequency of waterborne pathogens depending upon the conditions prevailing around the sources of water, protection of source of water, treatment and the wellbeing of supply system, thereby deteriorating the bacteriological state of waters and increasing the risk of transmission of various diseases.^13^,^14^

Analysis of 16S rRNA gene sequences of bacterial isolates revealed that most abundant sequence types were, *Bacillus cereus*, *Bacillus thuringiensis* and *Bacillus spp*. which belong to the family of *Bacillaceae*. The class of *Bacilli* was found in the water as a major pollution. The affiliation of the strains to the nearest phylogenetic neighbor and the percentage of 16S rRNA gene sequence similarities showed that bacterial strains are closely related to one another and exhibit 94 to 99% sequence similarity at the 16S rRNAgene sequence level.^15^,^16^

Pindi et al.^17^ reported similar results that phylogenetic analysis based on 16S rRNA gene sequences indicated that the 21 *Acinetobacter* strains belonged to 4 groups, 9 *Bacillus* strains belonged to 7 groups, 8 *Pseudomonas* strains belonged to 3 groups, 7 *Aeromonas* strains belonged to 5 groups, and 2 *Methylobacterium* strains formed two distinct groups^17^,^18^ which indicated the originality and authenticity of our study data.

## CONCLUSION

Among the 30 drinking water samples tested, 18 were found contaminated with bacteria. Molecular identification by 16s rRNA gene analysis of selected bacterial isolates showed the presence of pathogenic bacteria, *Bacillus spp*. The results of the current study are important for all from consumers to water supply companies. This suggests a great need that water supply companies should concentrate on this alarming presence of *Bacillus* bacteria in drinking water sources to avoid water pollution and provide safe drinking water. This also indicates the resistance potential of *Bacillus spp*. bacteria in present drinking water sources even after the standard precautionary measurement of water supply companies. Gene sequencing data generated in this study could be used to develop assays for the monitoring of potentially active pathogenic bacteria in drinking water systems, particularly for highly resistant pathogenic bacteria *Bacillus spp*. found as an alarming indication to be treated on priority bases. Water consumers should also take into account these findings during purchase and use of drinkable water.

### Authors’ contribution

***Neveen Hassan Eid and Dr. Munazza Gull*** conceived, designed and did statistical analyses and ***Dr. Huda Al Doghaither and Dr. Munazza Gull*** helped in manuscript writing and editing of manuscript while ***Dr. Taha Abdullah Kumosani*** reviewed and did final approval of manuscript.
